# Eyelid Lesion of Molluscum contagiosum: A Case Report and Literature Review

**DOI:** 10.7759/cureus.52272

**Published:** 2024-01-14

**Authors:** Sandhya Chaurasia, Varun Rastogi, Nisha Maddheshiya, Dilasha Dhungel, Karthikeyan Ramalingam

**Affiliations:** 1 Oral Pathology, Universal College of Medical Sciences and Teaching Hospital, Bhairahawa, NPL; 2 Oral Medicine and Radiology, Institute of Medical Sciences, Banaras Hindu University, Varanasi, IND; 3 Oral Pathology and Microbiology, Saveetha Dental College and Hospitals, Saveetha Institute of Medical and Technical Sciences, Saveetha University, Chennai, IND

**Keywords:** henderson-patterson bodies, lower eyelid, viral, treatment, pathogenesis, infection, nodule, eyelid, mollusca, molluscum contagiosum

## Abstract

Molluscum contagiosum (MC) is a common viral infection in children that affects the skin and oral mucous membranes. It is caused by the molluscum contagiosum virus (MCV), a double-stranded DNA virus in the *Poxviridae* family. Transmission takes place via direct skin contact, self-inoculation, and exposure to contaminated objects. Clinically, it is characterized by the presence of a single or multiple enlarged dome-shaped or doughnut-shaped flesh-colored papules with central umbilication, usually called "mollusca". The diagnosis of MC is based mainly on clinical observations, in addition to histopathological examinations to reveal characteristic molluscum bodies, also known as Henderson-Patterson bodies. Current treatment methods include mechanical, chemical, immune modulation, and antiviral treatments. In this context, we present a case involving a 42-year-old male infected with MC, outlining both the clinical and histopathological findings.

## Introduction

Molluscum contagiosum (MC) is a superficial skin infection caused by the molluscum contagiosum virus (MCV), which is categorized as a double-stranded DNA virus belonging to the *Poxviridae* family. MC accounts for only 1% of all skin diseases diagnosed worldwide. It is frequently seen in children, sexually active adults, and individuals with compromised immune systems. In addition, its prevalence is higher in tropical climates [1]. 

The prevalence of MC has been identified at 5-15% in immunocompetent children and approximately 8% in immunocompetent adults [2]. Each year, an estimated 8,000 cases per 100,000 individuals have been reported. According to a report from 2010, about 122 million individuals worldwide were affected by MC [3]. It typically manifests in areas, such as the arms, legs, trunk, and face, particularly affecting the eyelids. Clinically, it presents small flesh-colored bumps with a distinctive pearl doughnut shape and a central depression referred to as mollusca. The clinical appearance itself often leads to diagnosis, but histopathological examination can be employed to validate a diagnosis that has not been definitively established through clinical observations [4]. 

The histopathological examination of MC indicates an acanthotic epidermis that harbors intracytoplasmic molluscum bodies [5]. Possible mechanisms contributing to the development of acanthotic epithelium induced by the MCV include the upregulation of the epidermal growth factor receptor, vesicle anomalies, interference with cell-cycle regulation, or a combination of these factors. Dermatoscopy reveals peculiar features, such as a central pore with white-to-yellow amorphous polylobular structures and peripheral crown vessels. Under polarized light microscopy, these lesions exhibit rosette-like structures [6].

Practicing good hygiene and taking preventive measures are essential to minimize the risk of transmission in affected individuals and others. The lesions typically resolve on their own over time, but medical interventions may be considered for cosmetic reasons or if the lesions become irritated or infected. Treatment options include cryotherapy, laser therapy, curettage, imiquimod, and topical medications (cantharidin or podophyllotoxin cream) [7,8]. Here, we present a case study of a 42-year-old male afflicted with MC, outlining both the clinical and histopathological findings.

## Case presentation

**Figure 1:**
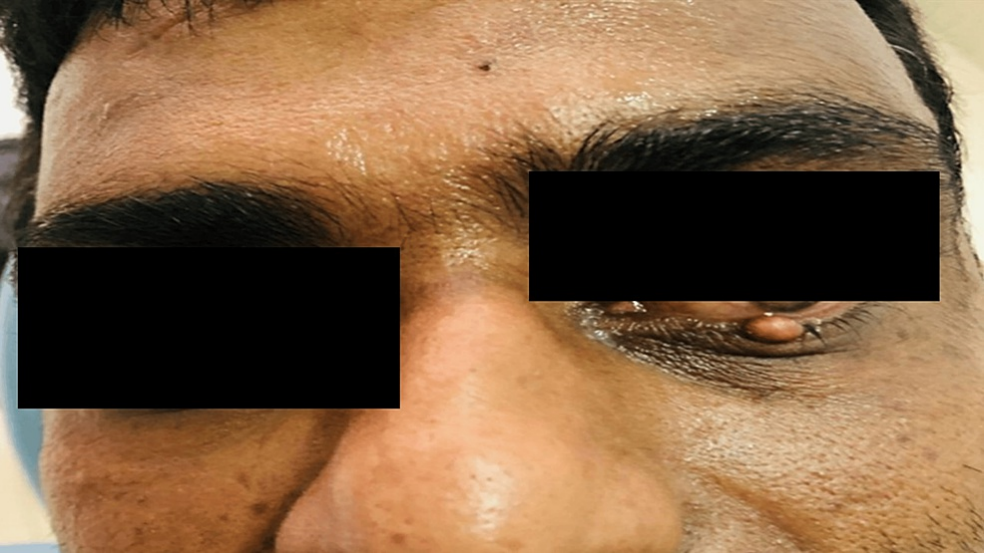
Clinical picture showing the nodular lesion on the left lower eyelid.

A 42-year-old male visited the Department of Oral Medicine and Radiology with a complaint of swelling in his left lower eyelid that had been present for the last 1.5 years with intermittent redness and excessive tears. His past medical history and surgical history were non-contributory. The habit history was not relevant and no immune disturbances. A clinical examination revealed a flesh-colored, dome-shaped nodule measuring about 1 cm x 0.5 cm in the left lower eyelid. It was a smooth lesion without any surface ulceration or discharge. The nodule was sessile and non-tender. There was mild erythema in the lower portion of the eyelid and sclera but no associated tenderness (Figure 1).

**Figure 2:**
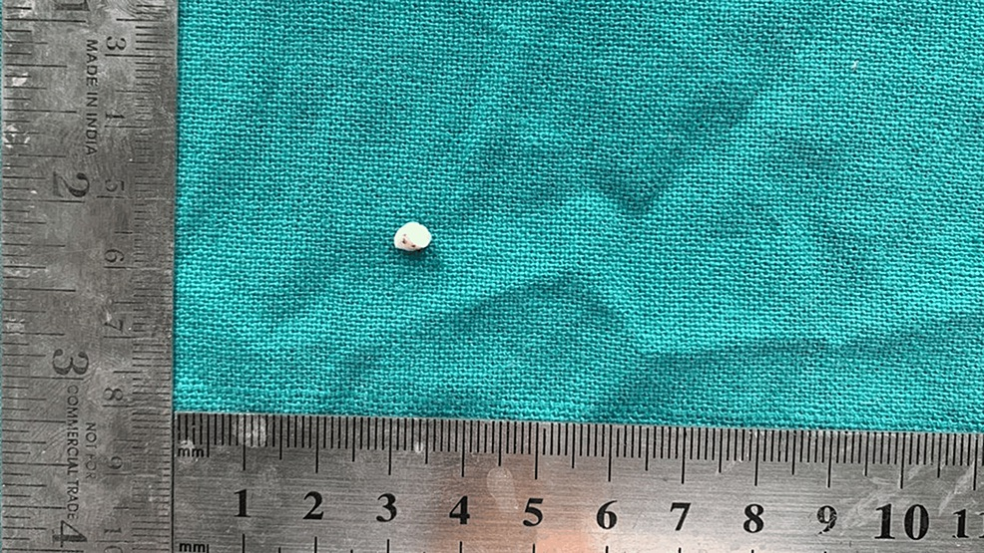
Grossing image of the excised specimen measuring 0.6 x 0.5 x 0.2 cm in dimension.

Routine blood tests did not reveal any abnormalities. Based on the clinical features observed, a provisional diagnosis of MC was established. Subsequently, the lesion was surgically excised under local anesthesia. Ibuprofen with paracetamol combination was given post-operatively for pain relief, if any. The biopsy specimen was preserved in 10% formalin, and the histopathological analysis was performed. Its surface displayed a smooth, pinkish appearance and was soft in consistency (Figure 2).

**Figure 3:**
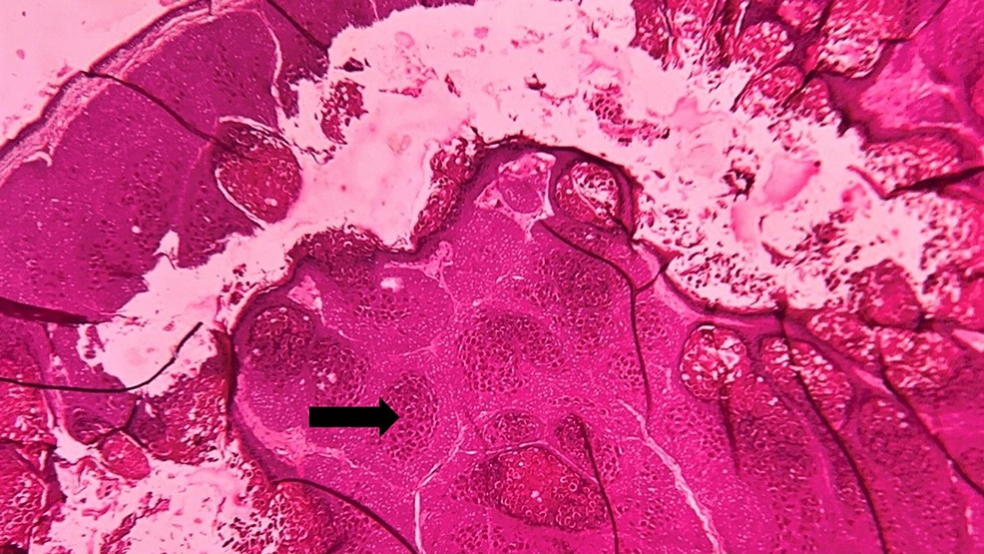
Photomicrograph showing the nodular lesion with cellular inclusions (black arrow) (hematoxylin and eosin (H&E), 4x)

The histopathological examination, conducted using routine hematoxylin and eosin (H&E) staining, revealed hyperplastic stratified squamous epithelium, resulting in a cup-shaped indentation or nodule (Figure 3).

**Figure 4:**
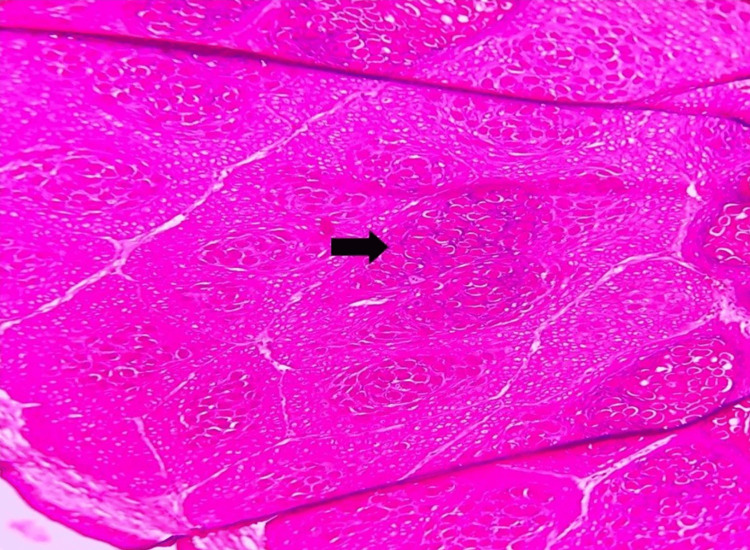
Photomicrograph showing the acanthotic epithelium with eosinophilic inclusions (black arrow) (hematoxylin and eosin (H&E), 10x)

In the epidermis, there is evidence of acanthosis, and the basal cell layer displays an enlarged basophilic nucleus along with the presence of mitotic figures. Keratinocyte cells in the spinous and granular layer showed amphophilic cytoplasm characterized by clear vacuolization (Figure 4).

**Figure 5:**
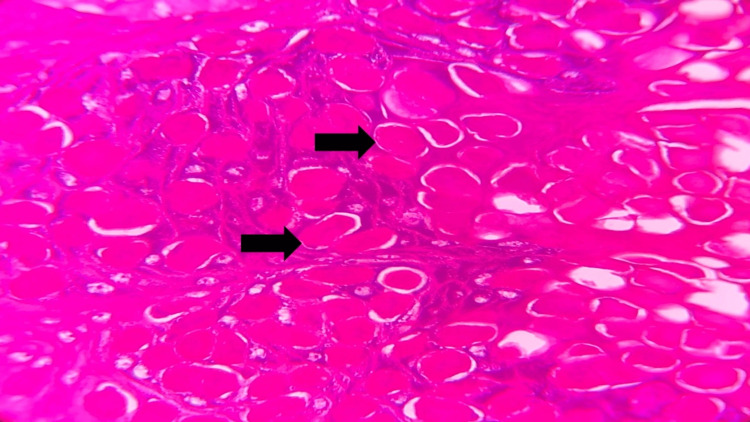
Photomicrograph showing eosinophilic inclusion bodies within the epithelial cells representative of Henderson–Patterson bodies (black arrow) (hematoxylin and eosin (H&E, 40x).

In addition, large intracytoplasmic basophilic granular viral inclusions, commonly referred to as Henderson-Patterson (H-P) or molluscum bodies were observed (Figure 5).

**Figure 6:**
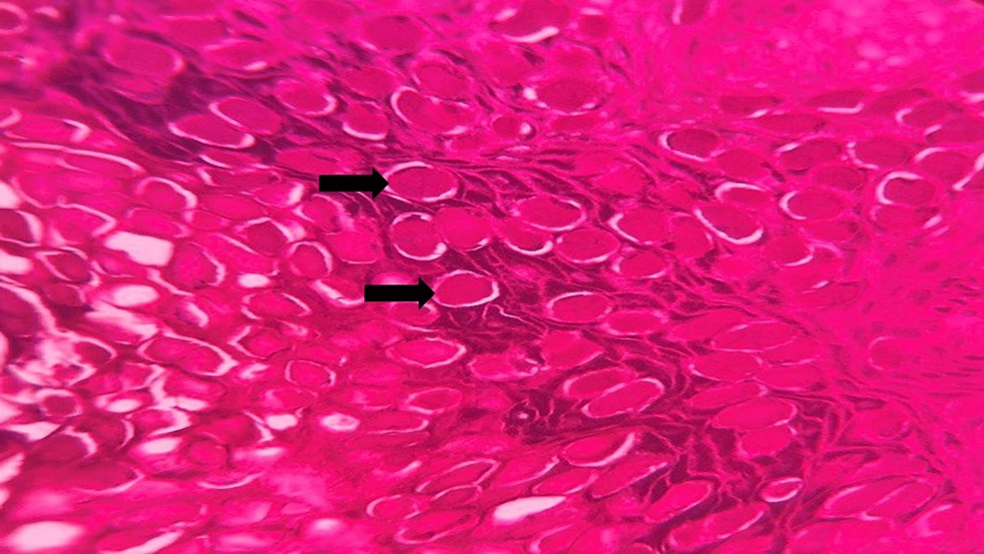
Photomicrograph showing eosinophilic inclusion bodies within the epithelial cells representative of Henderson–Patterson bodies (black arrows) (hematoxylin and eosin (H&E), 40x).

Some keratinocytes exhibited an eosinophilic cytoplasm, causing their nuclei to be displaced toward the periphery, resulting in a signet ring-like appearance (Figure 6).

Upon comparing the clinical and histopathological results, the diagnosis of MC was confirmed for the lesion. The patient was re-evaluated one month after the excision, and no signs of recurrence or scarring were observed in the surgical area.

## Discussion

**Figure 7:**
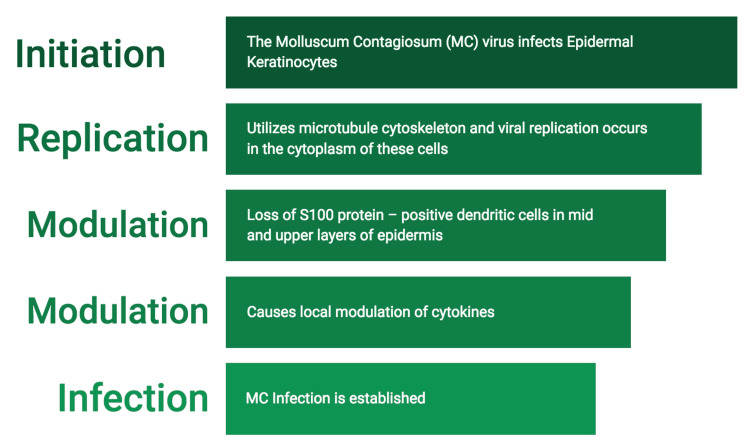
Picture showing the mechanism of viral replication Image credit: Dr. Karthikeyan Ramalingam

In 1817, Bateman provided the initial description of MC in the medical literature [9], while Juliusberg later identified the viral cause of the infection [10]. In particular, MCV is the largest infectious virus in the human body [11-12]. There are four types of MCVs: MCV I, MCV II, MCV III, and MCV IV. MCV I is the most prevalent, whereas MCV II is frequently found in adults [8,13]. Figure 7 explains the mechanisms of virus replication. It is a benign self-limiting childhood disease with an incubation time of two to seven weeks [14-15]. The risk factors for the spread of MC infection include direct skin-to-skin contact, autoinoculation, contaminated objects (e.g., towels, sponges, public baths, swimming pools, and tattoo instruments), sexually active adults, and immunocompromised patients, such as human immunodeficiency virus (HIV) infection [13-15].

MC usually occurs in children aged two to five. It commonly manifests on the extremities and face, especially on the eyelids, and oral lesions can occur on the lips, tongue, and mucous membranes in the mouth [4]. Sexually active adults and those with weakened immune systems (e.g., AIDS) often have lesions in the genital area, abdomen, and inner thighs [13,16]. Clinically, the MC lesion initiates as a small papule with a round or dome shape [17], and as the condition progresses, the lesions develop into larger, pearly, flesh-colored bumps called mollusca with a distinctive central depression, resembling a doughnut shape. The lesions exhibit a smooth surface and well-defined borders, and their color can range from pink to dark red, sometimes with hints of violet tones [1]. Table 1 summarizes the differential diagnosis of MC in HIV patients.

**Table 1:** Differential diagnosis and clinical presentation and differential features of related lesions

Condition	Clinical presentation	Differential features
Warts (verrucae)	Raised skin growths, rough surface	May have black dots (thrombosed capillaries)
Keratoacanthoma	Rapidly growing, dome-shaped nodule with a central crater	Rapid growth, central keratin plug
Condyloma acuminatum (genital)	Warty growths, often cauliflower-like appearance	Typically located in genital area
Kaposi’s sarcoma	Red or purple skin lesions, often on legs or face	Lesions are usually more vascular and may bleed
Basal cell carcinoma	Slow-growing, pearly papule with telangiectasia	Bleeding, ulceration, history of growth
Cryptococcosis	Papules or nodules with central umbilication	Often associated with systemic symptoms
Histoplasmosis	Lung nodules, fever, fatigue, chest pain	Ranges from asymptomatic to severe respiratory or disseminated infection

**Figure 8:**
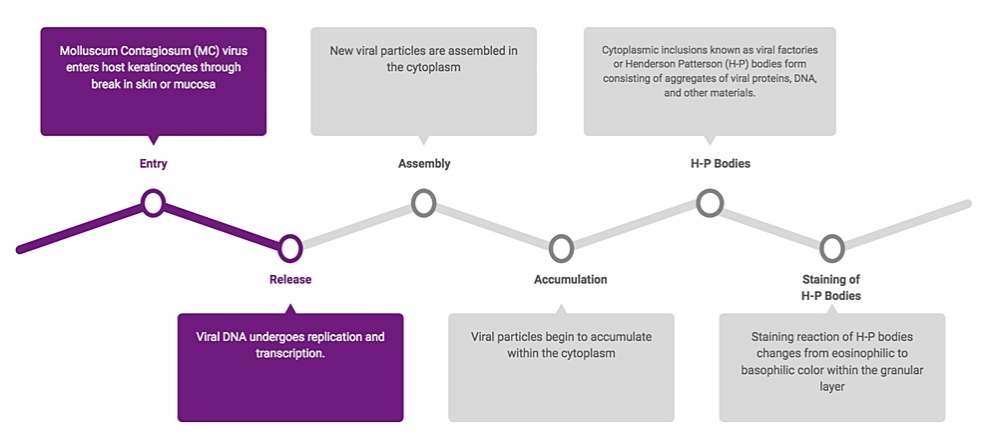
Formation of Henderson-Patterson bodies within the epithelial cells Image credit: Dr. Karthikeyan Ramalingam

Henderson and Patterson first documented the presence of intracytoplasmic inclusions called H-P or molluscum bodies in 1841. These inclusions are characterized by their large, ellipsoid shape and uniform distribution within the spinosum and granulosum layers of the infected epithelium. They have a basophilic appearance and measure approximately 25 micrometers in diameter [15,16,17]. Through electron microscopic investigations, it has been observed that these inclusions encase MCV within membrane-bound structures, often composed of aggregates of nuclear and cytoplasmic aggregates and proteins [4,15,18]. These inclusion bodies serve as the sites for viral replication [19]. The pathogenesis and mechanism of viral replication are summarized in Figure 7. The mechanisms of the formation of these H-P inclusion bodies are elucidated in Figure 8.

Due to its self-limiting nature, there is an ongoing debate regarding the necessity of active treatment for this condition. The research led by van der Wouden et al. demonstrated that no single intervention has been definitively proven to be effective in treating MC [15,18]. Treatment is recommended in cases where there are aesthetic concerns, secondary infections, coexisting skin conditions, or associated conjunctivitis [17-20]. An outline of the different treatment approaches can be found in Table 2.

**Table 2:** Various therapeutic options for molluscum contagiosum infection

Mechanical methods	Chemical methods	Immunomodulators	Antiviral medications	Newer techniques
Curettage	Cantharidin, topical retinoids	Imiquimod cream	Cidofovir cream	Topical sinecatechins
Cryotherapy	Potassium hydroxide	Oral cimetidine, diphencyprone	Trichloroacetic acid	Intralesional - 5-fluorouracil
Pulse dye laser therapy	Trichloroacetic acid	Interferon alfa, Candida antigen injection		Zoster immune globulin

## Conclusions

MC cases are primarily diagnosed based on clinical observations, with a key identifying feature being the central depression or umbilication of a dome-shaped lesion. However, histopathological analysis can serve as a valuable adjunct in confirming the diagnosis of MC by revealing the characteristic H-P bodies. The majority of these lesions are transmitted through direct skin-to-skin contact and are considered contagious. A prompt diagnosis by a dentist, followed by a timely referral to a dermatologist, can effectively impede the advancement of the disease.
